# New Appliances and Things Medical

**Published:** 1896-06-13

**Authors:** 


### NEW APPLIANCES AND THINGS MEDICAL.

[We shall be glad to receive, at our Office, 428, Strand, London, W.C., from the manufacturers, specimens of all new preparations and appliances, which may be brought out from time to time.]

### CHINOSOL, A NEW ANTISEPTIC AND DISINFECTANT.

(B. Kühn, 36, St. Mary at-Hill, Eastcheap, E.C.)

This antiseptic, which was discovered and prepared by Franz Fritzsche and Co., of Hamburg, is stated to be a product of the chinoline series. The latter bodies, which may be obtained as derivatives of cinchonine and quinine, are more advantageously prepared by the oxidation of nitrobenzine and aniline. They are powerful germicides and antiseptics, and can be taken in large doses with no injurious effect to the system. Chinosol, while possessing the general properties of the chinoline series, is stated to possess many additional advantages which should recommend it for use in surgery, gynæcology, dentistry, and medicine. According to the bacteriological report of W. Chattaway and T. H. Pearmain, its germicidal properties are exercised on the organisms of typhoid, diphtheria, cholera, and erysipelas in solutions, which for degree of dilution compare very favourably with antiseptics in general use, such as carbolic acid and perchloride of mercury. Since chinosol does not coagulate albumen, is extremely diffusible, and neither caustic nor toxic, it is an extremely valuable media for the antiseptic treatment of wounds or ulcerating surfaces. Chinosol may be obtained in powder, or in the form of tabloids, which are non-hydroscopic, and will keep indefinitely. Solutions prepared from these of the strength of 1 in 300 (1 tablet 15 grains, in 10 ounces of cold water) constitute useful antiseptic solutions. Greater degrees of concentration or dilution can be employed according to the object in view.

### ELECTROZONE AND MEDITRINA, NEW ANTISEPTICS AND GERMICIDES.

(British Electrozone Corporation, Limited, 1, Northumberland Avenenue, W.C.)

These new antiseptic preparations are manufactured from ordinary sea water, through which an electric current has been allowed to flow, in accordance with a method patented by the Electrozone Company. As a result of the electrolysis, part of the chloride, bromide, and iodide are stated to be converted into hypochlorites, hypobromites, and other compounds; and in meditrina there is said to be some 300 grains of "available chlorine" per gallon. For disinfecting purposes, for washing wounds, and for destroying bacteria wherever present, electrozone and meditrina are clearly well adapted. And if the experiments which are now being made at Maidenhead in the treatment of sewage effluent by means of this altered sea water prove as successful as they at present promise to be, electrozone will be one of the most valuable antiseptics. For household and domestic use as a sanitary disinfectant and deodoriser we are inclined to think electrozone will prove most useful.

